# The *Lancet* Sanitary Commission

**Published:** 1856-04

**Authors:** 


### THE "LANCET" SANITARY COMMISSION.†

The document before us is written in defence of Dr. Hassall, the well known and successful microscopist. We wish to take no partisan view on the question here discussed; but a careful perusal of the work only re-excites in us a regret we often feel, that in science the leaven of jealousy is ever fermenting. The facts of the present case seem simple enough. Mr. Wakley, the editor of the *Lancet,* originated an idea of investigating the subject of the adulteration of food on a grand scale. He placed the inquiry in the hands of Dr. Hassall, who conducted it with an accuracy of research which is deserving of the highest praise. In the course of the inquiry, Dr. Hassall very properly took chemical opinion, whenever it was required, of Dr. Letheby. When the labours of the inquiry were concluded, some friends of Dr. Hassall wished to present him with a testimonial, in recognition of his public services; upon which Mr. Wakley considered that any testimonial of this kind was due to him for having conceived the task, supplied the costs of its prosecution, and borne the probable legal responsibilities. This was natural enough; but the presentation of a testimonial to Mr. Wakley by his friends and admirers, need not have interfered with the presentation of a similar mark of respect to Dr. Hassall.

We deeply regret this controversy: it is humiliating to science generally. Dr. Hassall is only known to us by his published writings, but these we have ever read with interest

† The Correspondence relating to the *Lancet* Sanitary Commission Examined. By James Cæsar Benford, Esq., John A. Power, L.M., M.A., and Raymond S. Daniel, M.A. London: 1856.

and pleasure; and, on our parts, it required no such treatise as the one under notice to sustain the conviction that he is a man who has worked laboriously and usefully, and who deserves the recognition of his country much more than half the men in high places, who receive public thanks without a murmur, for doing those things which ought not to be done, for leaving undone those things which ought to be done, and for having no health in them.

